# Lsi2: A black box in plant silicon transport

**DOI:** 10.1007/s11104-021-05061-1

**Published:** 2021-07-10

**Authors:** Devrim Coskun, Rupesh Deshmukh, S. M. Shivaraj, Paul Isenring, Richard R. Bélanger

**Affiliations:** 1 Département de Phytologie, Faculté Des Sciences de L’Agriculture Et de L’Alimentation (FSAA), Université Laval, Québec, Québec Canada; 2 National Agri-Food Biotechnology Institute (NABI), Mohali, India; 3 CSIR-National Chemical Laboratory, Pune, India; 4 Département de Médecine, Faculté de Médecine, Université Laval, Québec, Québec Canada

**Keywords:** Silicon, Lsi2, Efflux, Membrane transport, Xylem loading, Root-to-shoot translocation

## Abstract

**Background:**

Silicon (Si) is widely considered a non-essential but beneficial element for higher plants, providing broad protection against various environmental stresses (both biotic and abiotic), particularly in species that can readily absorb the element. Two plasma-membrane proteins are known to coordinate the radial transport of Si (in the form of Si(OH)_4_) from soil to xylem within roots: the influx channel Lsi1 and the efflux transporter Lsi2. From a structural and mechanistic perspective, much more is known about Lsi1 (a member of the NIP-III subgroup of the Major Intrinsic Proteins) compared to Lsi2 (a putative Si(OH)_4_/H^+^ antiporter, with some homology to bacterial anion transporters).

**Scope:**

Here, we critically review the current state of understanding regarding the physiological role and molecular characteristics of Lsi2. We demonstrate that the structure–function relationship of Lsi2 is largely uncharted and that the standing transport model requires much better supportive evidence. We also provide (to our knowledge) the most current and extensive phylogenetic analysis of Lsi2 from all fully sequenced higher-plant genomes. We end by suggesting research directions and hypotheses to elucidate the properties of Lsi2.

**Conclusions:**

Given that Lsi2 is proposed to mediate xylem Si loading and thus root-to-shoot translocation and biosilicification, it is imperative that the field of Si transport focus its efforts on a better understanding of this important topic. With this review, we aim to stimulate and advance research in the field of Si transport and thus better exploit Si to improve crop resilience and agricultural output.

**Supplementary Information:**

The online version contains supplementary material available at 10.1007/s11104-021-05061-1.

## Introduction

The field of plant silicon (Si) biology has garnered tremendous attention, particularly in the past two decades. This can be attributed to the growing realization that, despite being considered a non-essential element (with the possible exception for the Equisetaceae), Si is now officially regarded as a beneficial element since it confers many plants with heightened resilience against environmental stress, both biotic and abiotic (Epstein 1994; 1999; Liang et al. 2015; Coskun et al. 2019a). The extent to which plants benefit from Si relies upon its accumulation in tissues, which typically varies from *c*. 0.1% to 10% (on a dry-weight basis), and displays strong cultivar, species, and larger phylogenetic differences (Epstein 1994; Ma 2004; Hodson et al. 2005; Trembath-Reichert et al. 2015; Coskun et al. 2019a; Deshmukh et al. 2020). Although the precise mechanistic properties of Si in plants remain elusive and contentious, it has recently been argued that contrary to current orthodoxy, the bioavailable form of Si (i.e., silicic acid (Si(OH)_4_)) has little if any intracellular role; rather, its deposition as silica (SiO_2_, via biosilicification; Belton et al. 2012; Kumar et al. 2020) within the extracellular matrix (particularly within the root endodermal and shoot tissues) acts simply as a protective agent against the numerous environmental stressors plants encounter (Coskun et al. 2019a, b).

From a wider perspective, the global biogeochemical cycling of Si, which is intricately coupled to the global carbon (C) cycle, is critically dependent on plants (Street-Perrott and Barker 2008; Struyf et al. 2009; Carey and Fulweiler 2012; de Tombeur et al. 2020; Tan et al. 2021). As Raven (2003) noted, the Poaceae (among the highest Si-accumulating angiosperm families) fix *c*. 15 Pg C per year out of *c*. 60 Pg C per year (i.e., *c*. 25%) of net primary production on land. Meanwhile, the decomposition of Si-accumulating plants returns Si to the oceans, where diatoms (for which Si is essential) fix > 15 Pg C per year out of *c*. 50 Pg C per year (> 30%) of net primary production in oceans.

**Fig. 1:**
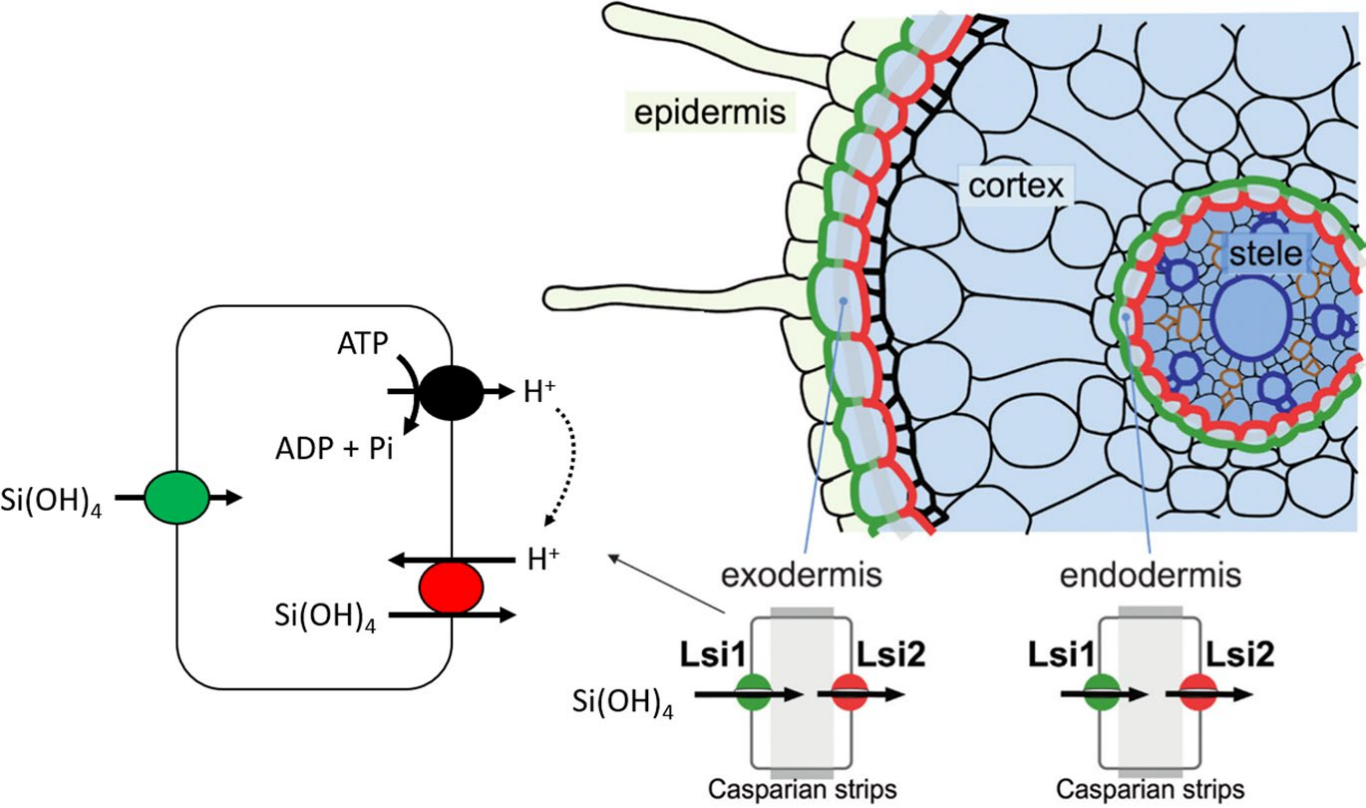
The standing Si-transport model in the roots of rice (*Oryza sativa*). Lsi1 and Lsi2 are expressed in the distal and proximal ends, respectively, of the exodermis and endodermis. Lsi1 mediates the thermodynamically passive uniport of Si(OH)_4_, whereas Lsi2 is thought to mediate the secondary active transport of Si(OH)_4_ in antiport with H^+^ (the electrochemical gradient of which is generated by the plasma-membrane H^+^-ATPase). Redrawn from Ma and Yamaji (2008)

In rice (*Oryza sativa* L.), two genes, *OsLsi1* and *OsLsi2* (*Low silicon 1* and *2*, named after the low Si content observed in tissues of the respective loss-of-function mutants), are known to dictate the radial transport of Si from soil to xylem through their presence in roots (Ma et al. 2006, 2007; Ma and Yamaji 2015). These genes code for plasma-membrane transporters that are proposed to act in coordination in the symplastic movement of Si to bypass apoplastic (Casparian band) barriers of the exodermis and endodermis. Specifically, OsLsi1 is expressed in the distal end of exodermal and endodermal cells and mediates the thermodynamically passive uptake of Si(OH)_4_, whereas OsLsi2 is expressed in the proximal end of the same cells and mediates the secondary active efflux of Si(OH)_4_ in exchange for protons (H^+^) (Ma and Yamaji 2015; Fig. 1). In other species where this model has been tested, Lsi1 can display a much wider expression profile (including in epidermal and cortical cell layers), whereas Lsi2 appears to be predominantly expressed in the endodermis (Table 1). In fact, thus far, only rice has been shown to display such specific polar and cellular localization of Lsi1 and Lsi2 and this feature has been proposed to explain the high accumulation of Si in shoot tissues (Mitani-Ueno and Ma 2020; see ‘Protein localization’ subsection, below).

**Table 1:** Literature survey of *Lsi1* and *Lsi2* gene expression responses to Si supplementation and associated protein (tissue and cellular) localization in roots of those species where both genes have been studied

Species	Gene	Gene expression response to Si	Protein tissue localization	Protein cellular localization	Reference
Rice (*Oryza sativa*)	*OsLsi1*	Downregulated^1^ Upregulated^2^	Basal root	Distal end of exodermis and endodermis	^1^Ma et al. 2006 ^2^Kim et al. 2014
	*OsLsi2*	Downregulated^1^ Upregulated^2^	Main and lateral roots (not in root hairs)	Proximal end of exodermis and endodermis	^1^Ma et al. 2007; Yamaji and Ma 2011 ^2^Kim et al. 2014
Maize (*Zea mays*)	*ZmLsi1*	Unaffected^1^ Downregulated^2^	Basal root	Distal end of epidermis, hypodermis, and cortex	^1^Mitani et al. 2009a ^2^Bokor et al. 2015
	*ZmLsi2*	Downregulated	Basal root	Endodermis (no polarity)	Mitani et al. 2009b; Bokor et al. 2015
Barley (*Hordeum vulgare*)	*HvLsi1*	Unaffected	Basal root	Distal end of epidermis and cortex, lateral root hypodermis	Chiba et al. 2009; Hosseini et al. 2017
	*HvLsi2*	Downregulated	Basal root	Endodermis (no polarity)	Mitani et al. 2009b; Hosseini et al. 2017
Pumpkin (*Curcurbita moschata*)	*CmLsi1*	n.d	Throughout root	Throughout root (no polarity)	Mitani et al. 2011
	*CmLsi2*	n.d	Throughout root	n.d	Mitani-Ueno et al. 2011
Cucumber (*Cucumis sativus*)	*CsLsi1*	Downregulated	Root tips	Epidermis and cortex (no polarity), distal end of endodermis	Sun et al. 2017
	*CsLsi2*	Upregulated	n.d	Endodermis (no polarity)	Sun et al. 2018
Horsetail (*Equisetum arvense*)	*EaLsi1* (*EaNIP3;1*)	Unaffected (in transformed *Arabidopsis thaliana*)	n.d	n.d	Grégoire et al. 2012
	*EaLsi2*	Unaffected	n.d	n.d	Vivancos et al. 2016
Tomato (*Solanum lycopersicum*)	*SlLsi1*	Unaffected	Throughout root	Throughout root (no polarity)	Sun et al. 2020
	*SlLsi2*	Unaffected	n.d	n.d	Sun et al. 2020

n.d., not determined

While much is known about the protein structure, transport function, and physiological role of Lsi1, the same cannot be said of Lsi2 in these regards. The influence of Lsi1 on tissue Si content is also better known than that of Lsi2. Yet, understanding Lsi2 is critical given that it appears to influence all downstream processes, from root-to-shoot translocation to biosilicification.

Here, we critically review the current state of understanding regarding the phylogeny, protein structure, functional determinants, transport mechanism, and physiological role of Lsi2. Throughout, we discuss several important but hitherto underexplored research questions: What is the role of Lsi2 and is it indeed necessary for Si accumulation *in planta*? What is the expression profile of *Lsi2*? And what is the mechanism of transport of Lsi2 and how is it regulated? We argue that much remains to be learned, especially when compared to Lsi1, and that many of the ideas proposed after the initial characterization of Lsi2 have not been subsequently challenged in the literature. If we are to effectively exploit Si to improve plant resilience to environmental stress, particularly in the context of the agricultural response to climate change, it is important that we better understand the fundamental mechanisms of Si acquisition in plants, including the role of Lsi2.

## Lsi1: A juxtaposition

Before discussing Lsi2, a brief review of Lsi1 is warranted to highlight the vast discrepancy in understanding between the two transporters. The influx channel Lsi1 is a member of the Nodulin26-like intrinsic protein-III (NIP-III) subgroup of the Major Intrinsic Proteins (MIPs; also known as aquaporins) which mediates the thermodynamically passive transport of Si(OH)_4_, as well as other metalloids, including boron (as B(OH)_3_) and arsenic (as As(OH)_3_) (Ma et al. 2006; 2008; Mitani-Ueno et al. 2011). First described in rice, OsLsi1 (OsNIP2;1) was found to be expressed predominately on the distal side of exodermal and endodermal cell plasma membranes in root cross-sections, and the basal portion of seminal and lateral roots, longitudinally (Ma et al. 2006; Yamaji and Ma 2007). Since then, *OsLsi1* homologs have been cloned and characterized from several species, including maize (*Zea mays*; Mitani et al. 2009a), barley (*Hordeum vulgare*; Chiba et al. 2009), pumpkin (*Curcurbita moschata*; Mitani et al. 2011), wheat (*Triticum aestivum*; Montpetit et al. 2012), horsetail (*Equisetum arvense*; Grégoire et al. 2012), soybean (*Glycine max*; Deshmukh et al. 2013), poplar (*Populus trichocarpa*; Deshmukh et al. 2015), cucumber (*Cucumis sativus*; Sun et al. 2017), tobacco (*Nicotiana sylvestris*; Coskun et al. 2019c), date palm (*Phoenix dactylifera*; Bokor et al 2019), grape (*Vitis vinifera*; Noronha et al. 2020), and tomato (*Solanum lycopersicum*; Deshmukh et al. 2015; Sun et al. 2020).

Interestingly, *OsLsi1* expression was also found to be downregulated in rice in response to several days of Si supplementation in the rooting media (Ma et al. 2006). However, this response appears to vary across other species. In addition, the patterns of cellular/tissue localization have also been seen to differ across plants. As Table 1 shows, *Lsi1* and *Lsi2* expression can be either suppressed, enhanced, or unaffected by Si, depending on the species; similarly, protein localization can vary widely.

Like all members of the MIPs, Lsi1 is composed of a tetramer with each subunit composed of six transmembrane domains and two half-transmembrane helices protruding from opposite ends towards the centre of the pore where a constriction site composed of two conserved NPA (Asp-Pro-Ala) domains is formed (Murata et al. 2000; Pommerrenig et al. 2015). A second constriction site, termed the selectivity filter, is composed of four amino acids, and is involved in solute specificity for given MIP subgroups (Hove and Bhave 2011).

For Lsi1, the selectivity filter is composed of a conserved GSGR (Gly-Ser-Gly-Arg) motif that confers selectivity for Si (Mitani-Ueno et al. 2011). The horsetail EaLsi1 (a NIP-II member) is an exception, with a selectivity filter composed of a STAR (Ser-Thr-Ala-Arg) motif (Grégoire et al. 2012). Deshmukh et al. (2015) noticed that some species, such as tomato, possess an Lsi1 channel, but nevertheless accumulate little Si in their tissues (*c*. 0.2%), and attributed this peculiarity to an extra amino acid (aa) between the NPA domains (i.e., 109 instead of the common 108 found in Si-accumulating species) which renders the channel much less permeable to Si. Sun et al. (2020) reported SlLsi1 from tomato to be Si-permeable (and attributed the low-Si phenotype *in planta* to a Si-impermeable Lsi2) but the rates of influx were indeed much lower than what is typically seen for Lsi1s from Si-accumulating species (e.g., rice, barley, soybean, etc.), suggesting perhaps the relationship between Lsi1 function and tissue accumulation is one of degree (see also Coskun et al. 2019c). Coskun et al. (2019c) recently described yet another exception in the case of NsLsi1 from tobacco, in which it possessed both a GSGR selectivity filter and 108 aa spanning the NPA domains, and yet displayed little Si permeability, matching the low-Si phenotype *in planta*. Here, a single aa substitution to a highly conserved residue found in Si-permeable homologs (i.e., P125F) resulted in a gain of function that coincided with increased plasma-membrane localization of the protein. A very similar scenario was also described for a Lsi1 variant from pumpkin (Mitani et al. 2011).

Taken together, these findings demonstrate a highly detailed and continuously developing description of the structure–function and genotype–phenotype relationships related to Lsi1, proving to be instrumental in understanding why species can and cannot accumulate Si (Coskun et al. 2019a, b; Deshmukh et al. 2020). However, as we shall describe next, this level of understanding does not currently apply to Lsi2.

## Lsi2: The black box

### Background

As with the discovery of *OsLsi1*, *OsLsi2* was identified by selecting a mutant rice plant that did not display deleterious symptoms when grown in the presence of germanium (Ge), a toxic analog of Si (Ma et al. 2007). This mutant was also shown to accumulate lower quantities of Si than the WT counterpart and exhibited lower grain yield when grown in the field (presumably because of reduced stress resistance). Genome mapping led to the identification of a locus that included the gene for a putative anion transporter of unknown function but shared some homology with the prokaryotic arsenite (As(III)) transporter, ArsB (Meng et al. 2004). Complementation of the mutant with the *OsLsi2* gene rescued the phenotype. Like *OsLsi1*, *OsLsi2* expression was shown to be downregulated in response to prolonged Si supplementation (Table 1). Moreover, OsLsi2 was found to localize within the plasma membrane at the proximal side of exodermal and endodermal cells (i.e., across from OsLsi1 in the same cell types). Heterologous expression of *OsLsi2* in the *Xenopus laevis* oocyte system revealed some Si-efflux activity in preloaded cells, but no influx activity in the experimental setup chosen.

Based on these results, it was suggested that OsLsi2 acted as an efflux transporter, and in conjunction with OsLsi1, transported Si across the root and into the stele (Fig. 1; Ma et al. 2007; Ma and Yamaji 2015). Since then, homologs have been cloned and characterized from barley (Mitani et al. 2009b), maize (Mitani et al. 2009b), pumpkin (Mitani-Ueno et al. 2011), horsetail (Vivancos et al. 2016), soybean (Bélanger et al. 2016), cucumber (Sun et al 2018), and tomato (Sun et al. 2020).

### Phylogeny

A recent phylogenetic analysis of silicon transporters across the biological kingdoms found *Lsi2* (or *Lsi2-like*) genes present throughout the eukaryotic supergroups, including Amoeboza, Opisthokonts (metazoa, sponges, and choanoflagellates), Haptophytes, Alveolates, Rhizaria, Stramenopiles (diatoms, chrysophytes), and the Archaeplastida (embryophytes) (Marron et al. 2016). Given that Si(OH)_4_ autopolymerizes into SiO_2_ above concentrations of *c*. 2 mM under most conditions (with the apparent exception of the xylem apoplast; see below; Belton et al. 2012) and free SiO_2_ in the cytoplasm is catastrophic for cellular metabolism (Iler 1979; Martin-Jézéquel et al. 2000; Montpetit et al. 2012), it has been proposed that siliceous organisms such as diatoms sequester Si within vesicles for controlled polymerization. Thus, Marron et al. (2016) proposed that the original function of Si-permeable transporters was as a detoxification mechanism to remove excess Si(OH)_4_ (or perhaps other toxic metalloids, such as As(OH)_3_) from the cytosol. Accordingly, Lsi2 may have had such a role very early in land plant evolution, even before the recruitment of NIP-III proteins (Lsi1) as passive Si channels some 500 Ma (million years ago; Trembath-Reichert et al. 2015; Pommerrenig et al. 2020; Deshmukh et al. 2020).

**Fig. 2:**
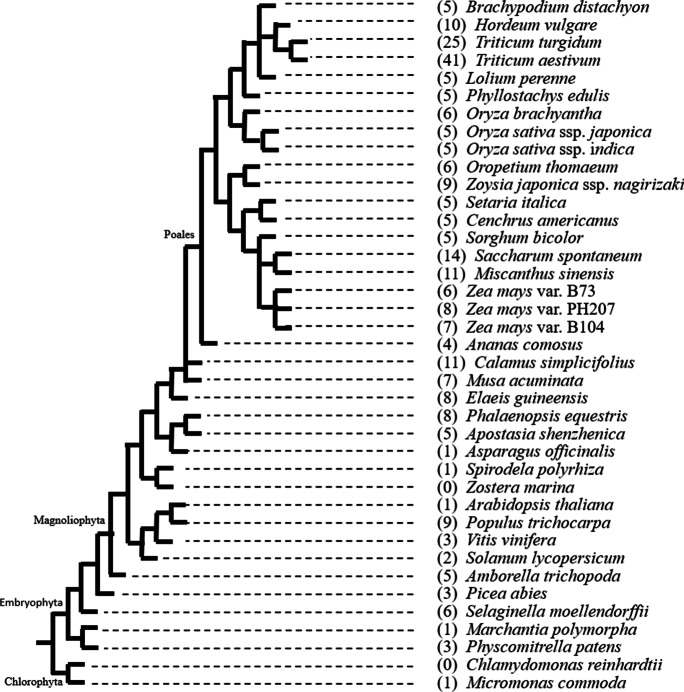
Taxonomical distribution of genome-sequenced plant species obtained from the PLAZA 4.5 database (Van Bel et al. 2018). Numbers in parentheses denote the number of Lsi2 homologs identified in each species (sequences provided in Supplementary Table S4

Our own phylogenetic analysis of *Lsi2* throughout the plant kingdom shows a high level of conservation throughout the embryophytes (bryophytes and tracheophytes) and presence even among the chlorophytes (green algae) (Fig. 2). Embryophytes are estimated to have evolved about 515 to 470 Ma (Morris et al. 2018), and the presence of *Lsi2* in primitive land plants indicates its early evolution. Interestingly, of the 37 embryophyte species studied, *Lsi2* was absent in only one, the seagrass *Zostera marina*. In fact, *Z. marina* is known to have lost entire repertoires of genes, including stomatal genes, genes involved in terpenoid synthesis and ethylene signalling, as well as genes for ultraviolet protection and far-red-sensing phytochromes (Olsen et al. 2016); whether *Lsi2* was similarly lost remains speculative. Likewise, it would appear that *Lsi2* is absent from the genome of the chlorophyte *Chlamydomonas reinhardtii* (Fig. 2).

**Fig. 3:**
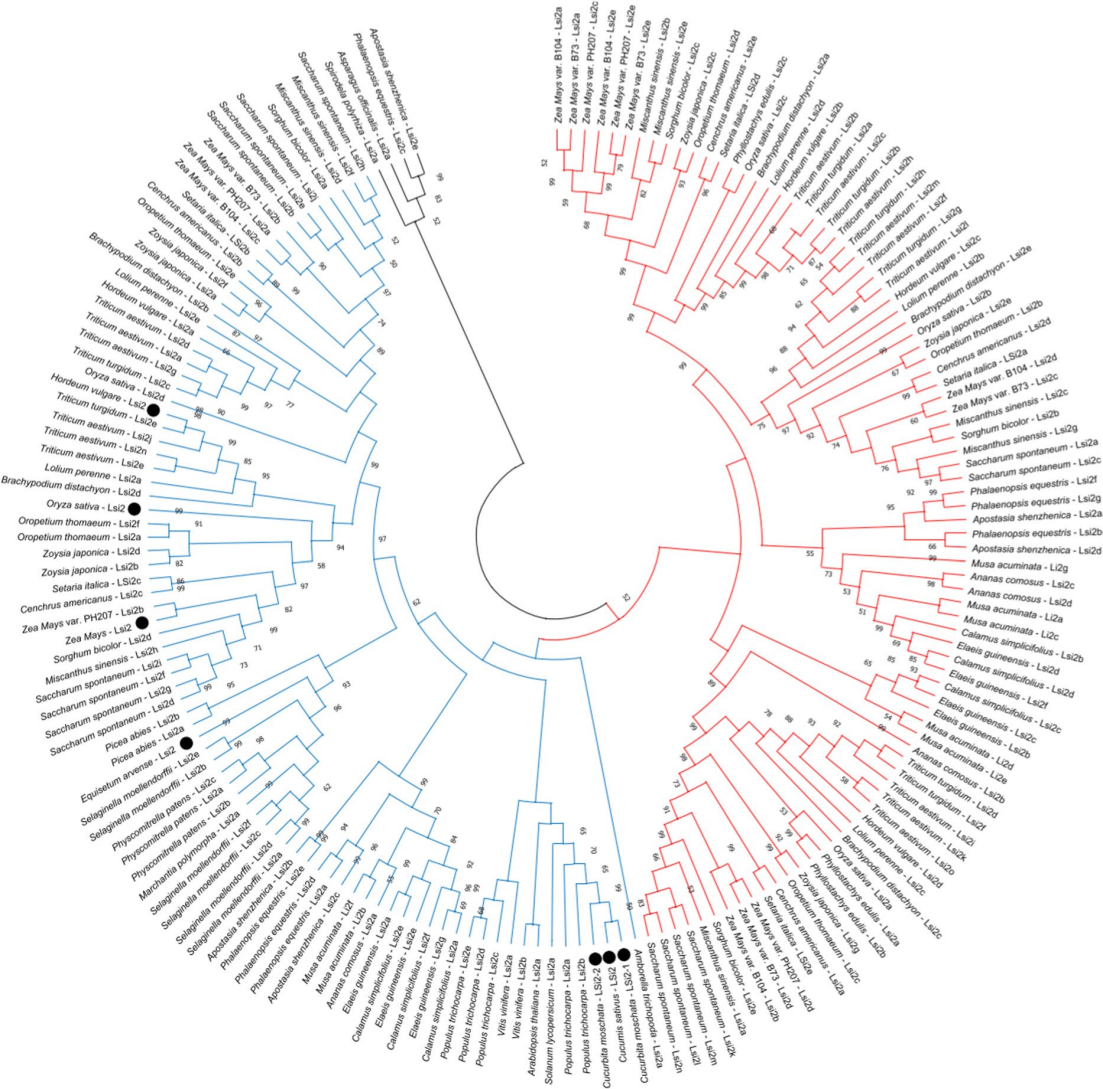
Phylogenetic distribution of Lsi2 homologs identified in Fig. 2, as well as those found in the species *Cucumis sativus*, *Cucurbita moschata*, and *Equisetum arvense* (which were not available in the PLAZA 4.5 database). To date, only seven sequences have been functionally characterized for Si transport (as denoted by the black circles; see text for details). The tree was developed using the maximum-likelihood method provided in MEGA 7. Only proteins > 400 amino acids were considered in the analysis. Sequences fell into three distinct clades, highlighted by blue, red, and black branches. Gene identifications can be found in Supplementary Table S3 and sequences are provided in Supplementary Table S4

A thorough phylogenetic analysis was further conducted on the Lsi2 homologs identified in Fig. 2, with the addition of those found in three other species (cucumber, pumpkin, and horsetail, which were not identified in the original genome database consulted for Fig. 2 but were included based on literature precedence; see ‘Background’ subsection, above). Thus, the tree was generated using 176 sequences that included all seven functionally characterized Lsi2s (Fig. 3). We found the sequences clustered into three major clades (denoted in black, blue, and red, and containing 4, 85, and 87 sequences, respectively). All functionally characterized Lsi2s grouped under a single clade (highlighted in blue; Fig. 3), indicating the higher sequence similarity among them. Moreover, these results highlight both the potential complexity and diversity of Lsi2 transporters and our limited knowledge of their properties and functionality and how they relate within and among clades.

### Protein structure

**Fig. 4:**
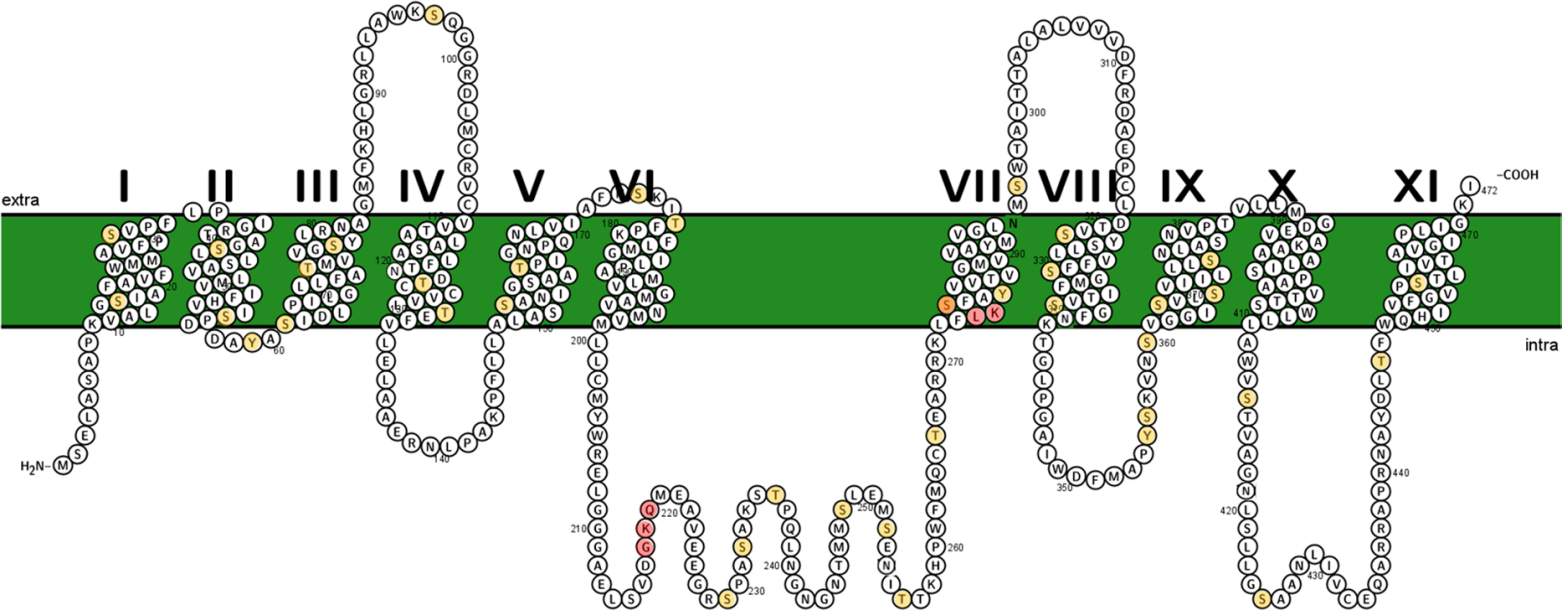
Predicted secondary structure of OsLsi2 from rice (*Oryza sativa*). Transmembrane domains have been labeled in Roman numerals. Highlighted red, the residues that show similarity to the GXQ motifs thought to underlie the Si-selectivity of diatomic SIT transporters (Knight et al. 2016; see text for details; see also Supplementary Fig. S3). Note, no evidence currently exists to support their involvement in Lsi2-mediated Si transport. Highlighted yellow, residues predicted to be able to be phosphorylated; see Supplementary Fig. S4 for details). Structure prediction based on the SOSUI algorithm (http://harrier.nagahama-i-bio.ac.jp/sosui/sosui_submit.html)

As Ma et al. (2007) first described, OsLsi2 consists of a 472-residue protein that is predicted to form 11 transmembrane domains flanked by an intracellular N-terminus and extracellular C-terminus (Fig. 4). As it stands, no crystal structures of Lsi2 proteins have been reported. However, a homology-based structural prediction reveals the bacterial Na^+^-dependent citrate transporter, NaCT (also known as SLC13A5; PDB ID: 4F35; Mancusso et al. 2012), to share 99.5% structural similarity with OsLsi2, albeit with very low sequence identity (17% overall; Supplementary Fig. S1) and 23.4% in 184 overlapping residues (Supplementary Table S1). Based on structural similarity, one could speculate that Lsi2 can transport Na^+^ and/or tricarboxylates such as citrate and dicarboxylates such as succinate, malate, and fumarate (Mancusso et al. 2012). In this regard, *in-silico* analysis indeed predicts citrate as the highest probability ligand for OsLsi2 (Supplementary Fig. S2). To our knowledge, no studies to date have investigated or reported transport of these potential substrates by Lsi2.

**Fig. 5:**
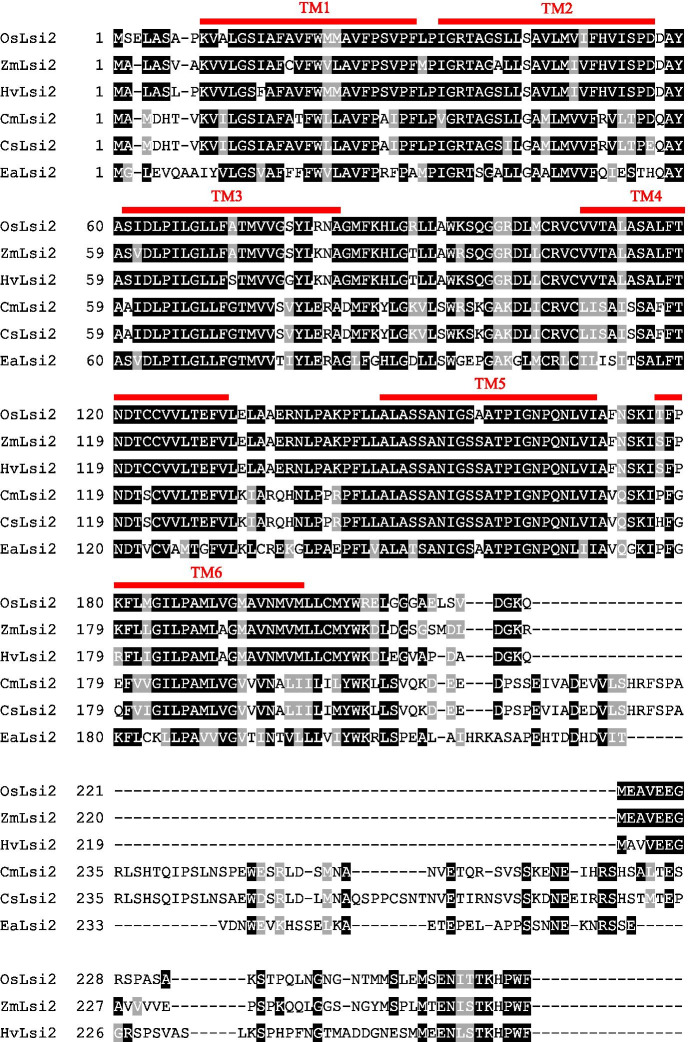

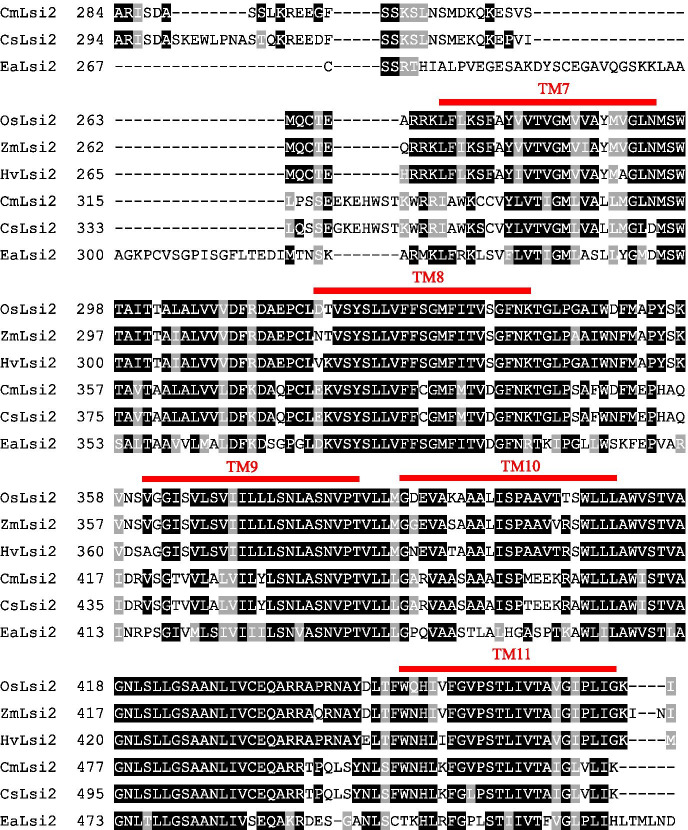
Sequence alignment of six Lsi2 proteins (OsLsi2, ZmLsi2, HvLsi2, CmLsi2-1, CsLsi2, and EaLsi2, from rice (*Oryza sativa*), maize (*Zea mays*), barley (*Hordeum vulgare*), pumpkin (*Curcurbita moschata*), cucumber (*Cucumis sativus*), and horsetail (*Equisetum arvense*), respectively) that have, to date, been functionally verified to transport Si (in the *Xenopus* oocyte system; see text for details). In red, the 11 predicted transmembrane (TM) domains

Protein sequence alignment studies of six Lsi2s that have, to date, been functionally identified as Si-permeable transporters in the *Xenopus laevis* oocyte system (i.e., OsLsi2, ZmLsi2, HvLsi2, CmLsi2-1, CsLsi2, and EaLsi2, from rice, maize, barley, pumpkin, cucumber, and horsetail, respectively) show a high degree of conservation among the transmembrane domains, and a very high degree of variability in between, particularly between transmembrane domains (TM) 6 and TM7, i.e., in the largest intracellular loop (Fig. 5). Interestingly, a few differences were observed between monocots and dicots, namely, a shorter and more hydrophobic TM6-TM7 loop for monocots (Supplementary Table S2). Whether these properties are germane to Si transport, and whether they relate to the relatively higher Si-accumulating properties generally observed among monocots (specifically the Poaceae), has yet to be determined.

The rat (*Rattus norvegicus*) SLC34A2 protein (also known as NaPiIIb), a mammalian sodium-phosphate cotransporter, was recently identified as a Si-efflux transporter that was upregulated in rat kidneys following chronic dietary Si deprivation (Ratcliffe et al. 2017). The authors noted that Si efflux was independent of extracellular HPO_4_^2−^ and Na^+^ concentrations. Noticeably, SLC34A2 shows very little similarity to SLC13A5 (23.6% identity in 55 overlapping residues) and OsLsi2 (40% in 20 overlapping residues; Supplementary Table S1).

The diatom silicic acid transporters (SITs) are the best described *bona-fide* Si transporters to date. For instance, CfSIT1 from the marine diatom *Cylindrotheca fusiformis* was first identified by Hildebrand et al. (1997). The SITs are ubiquitous among the diatoms and are upregulated during silicification and in response to Si(OH)_4_ limitation (see Shrestha and Hildebrand 2015, and references therein). Saturable Si(OH)_4_-transport kinetics via SITs have been demonstrated in isolated diatom membrane vesicles, *Xenopus laevis* oocytes, and reconstituted proteoliposomes, and have been shown to be dependent on the transmembrane electrochemical Na^+^ gradient (in a symport mechanism), likely in a 1:1 Na^+^/Si(OH)_4_ stoichiometry (see Knight et al. 2016, and references therein).

Like SLC13A5 and SLC34A2, very little similarity exists between SITs and Lsi2s, with only 23.5% sequence similarity in 34 overlapping residues between OsLsi2 and PtSIT1 from *Phaeodactylum tricornutum* (Supplementary Table S1). A key feature thought to be integral to Si(OH)_4_ binding in SITs is four highly conserved GXQ motifs that occur in pairs at the cytoplasmic ends of TM 2 and 3 and the extracellular ends of TM 7 and 8 (Knight et al. 2016). Interestingly, sequence alignment with OsLsi2 reveals the latter to indeed share the first GXQ motif (albeit within the TM 6–7 loop; i.e., GKQ at positions 218–220), some structural similarity with the second motif (LKS at positions 274–276), but no overlap with the second GXQ pair (Fig. 4; Supplementary Fig. S3). Further studies are needed to determine if these motifs are indeed involved in the Si-selectivity of Lsi2.

Lastly, an *in-silico* analysis predicting potential residues for phosphorylation (a key post-translational regulatory mechanism) identified 38 amino acids of interest for OsLsi2 (Fig. 4; Supplementary Fig. S4). Whether these residues are involved in protein phosphorylation and Lsi2 function remain to be evaluated.

### Protein function

Aside from studies investigating the responses to genetic modifications *in planta* (e.g., *Lsi2* knock-down, knock-out, or over-expression; Ma et al. 2007; Mitani et al. 2009b), to the best of our knowledge, the function of Lsi2 and its transport characteristics have thus far only been investigated in the *Xenopus laevis* oocyte expression system. Ma et al. (2007) first demonstrated Lsi2-mediated Si efflux after manually injecting Lsi2-expressing oocytes with a ^68^Ge-labelled Si(OH)_4_ solution and monitoring the release of the radioisotope into a Si-/Ge-free medium over several hours. Using this method, they measured an efflux activity of *c*. 1 pmol oocyte^−1^ h^−1^. They also demonstrated the flux to be pH-sensitive (decreasing with external alkalinity), temperature-sensitive (*c*. 80% reduction at 10 °C relative to 18 °C), and inhibited by the protonophores DNP, CCCP, and FCCP (*c*. 20 – 40% reduction from controls).

These observations led the authors to propose a secondary active Si(OH)_4_/H^+^ antiport mechanism (Fig. 1). Indeed, this may be intuitive, as secondary active transport in higher plants is predominately energized by H^+^ gradients (Pedersen and Palmgren 2017), as opposed to Na^+^ gradients (Sze et al. 1999; Mulkidjanian et al. 2008), which could explain the lack of Na^+^-powered SITs in this lineage (Marron et al. 2016). Since Ma et al. (2007), however, only a handful of studies have repeated these experiments, demonstrating Lsi2-mediated efflux for maize (Mitani et al. 2009b), barley (Mitani et al. 2009b), horsetail (Vivancos et al. 2016), and cucumber (Sun et al. 2018). Sun et al. (2020) studied SlLsi2 from tomato and concluded it was Si-impermeable. It is worth noting that, of all these studies, only Sun et al. (2018) repeated the temperature-sensitivity test described above. Otherwise, it would appear no other studies have conducted the system tests of Ma et al. (2007) (Table 2).

**Table 2:** Literature survey of the functional analyses of plant Lsi2 transporters. Note, all studies have been conducted in the *Xenopus laevis* oocyte system

Protein	Species	Si flux	Methodological notes	Reference
OsLsi2	Rice (*Oryza sativa*)	2 pmol oocyte^−1^ h^−1^ (pH_ext_ = 6) 1.5 pmol oocyte^−1^ h^−1^ (pH_ext_ = 7) 0.5 pmol oocyte^−1^ h^−1^ (pH_ext_ = 8)	50 nL injection of ^68^Ge-labelled Si solution (1 mM) ≤ 8-h efflux pH-sensitive flux DNP (0.5 mM), CCCP (10 μM), and FCCP (10 μM) inhibition Low-temperature (10 °C) inhibition	Ma et al. 2007
HvLsi2	Barley (*Hordeum vulgare*)	5% “efflux activity” ^a^	50 nL injection of ^68^Ge-labelled Si solution (1 mM) 4-h efflux	Mitani et al. 2009b
ZmLsi2	Maize (*Zea mays*)	1.6% “efflux activity”	50 nL injection of ^68^Ge-labelled Si solution (1 mM) 4-h efflux	Mitani et al. 2009b
CmLsi2-1, CmLsi2-2	Pumpkin (*Curcurbita moschata*)	15–30% “efflux activity”	50 nL injection of ^68^Ge-labelled Si solution (1 mM) 4-h efflux	Mitani-Ueno et al. 2011
EaLsi2-1	Horsetail (*Equisetum arvense*)	15–20% “efflux activity”	Oocytes injected with 25 nL Si solution (1 mM; control) or Si and Lsi2 cRNA and incubated for 72 h	Vivancos et al. 2016
CsLsi2	Cucumber (*Cucumis sativus*)	24 pmol oocyte^−1^ h^−1^	50 nL injection of 1 mM Ge solution 30-min efflux Low-temperature (4 °C) inhibition	Sun et al. 2018
SlLsi2	Tomato (*Solanum lycopersicum*)	No efflux activity detected	50 nL injection of 1 mM Ge solution 30-min efflux	Sun et al. 2020

^a^ Efflux activity = % released relative to amount injected into oocytes

Although the studies described above have provided useful basic information on Lsi2 functionality, they also raise several critical issues:

1. It remains disputable whether Ge (either as a stable or radioactive isotope) is a suitable proxy for Si in this system. Indeed, Ge has long been used as a proxy for studying Si in biological systems (Azam and Volcani 1981), including in SIT-expressing *Xenopus* oocytes (Hildebrand et al. 1997). Moreover, an absence of discrimination between ^68^Ge and Si has been demonstrated *in planta* for several plant species (Nikolic et al. 2007). Nevertheless, it remains unclear if Ge fluxes reliably capture Si fluxes in Lsi2-expressing oocytes due to a lack of kinetic analyses and competition assays between the two substrates (see e.g., Hildebrand et al. 1997). Incidentally, methods for direct measurements of Si fluxes have been developed to a high degree of sensitivity and reproducibility (Deshmukh et al. 2015; Garneau et al. 2015; 2018; Vivancos et al. 2016; Coskun et al. 2019c), which should eliminate the need for analogs altogether, at least under non-steady-state conditions such as described above, and provide a more reliable assessment of Si transport, contrary to arguments made by others (Sun et al. 2020).
2. Manual injection of Ge/Si into oocytes is a crude and potentially problematic procedure. For one, such non-steady-state conditions preclude an accurate appraisal of starting conditions for efflux assays. Wide variations in internal concentrations are inevitable across cells during the course of Si/Ge injections and transfers to efflux solutions. It is also unclear what a bolus of Si (and a chemically “temperamental” one, at that; see ‘Phylogeny’ subsection, above) does to the internal biochemistry of the cell. Lastly, the conclusion that Lsi2s cannot mediate Si uptake in the oocyte system is perhaps premature, as it is based on less than 2-h uptake assays (see Fig. S11 in Ma et al. 2007; see also Sun et al. 2018; 2020). Whether Lsi2-expressing oocytes could indeed be capable of Si uptake given sufficient time should be further investigated.
3. In its current application, the bioassay generates a flux occurring *down* a concentration gradient, i.e., a thermodynamically passive flux (one of facilitated diffusion). Although this does not necessarily preclude the possibility of a (secondary) active transport mechanism (i.e., an energy-dependent flux *against* a(n) (electro)chemical gradient), as the standing model suggests, this has yet to be experimentally demonstrated.
4. The Si(OH)_4_/H^+^ antiport model has also not been directly demonstrated (Ma and Yamaji 2008) (nor has an As(OH)_3_/H^+^ antiport model, for that matter; Lindsay and Maathuis 2017), as no electrophysiological analyses have been carried out to show that Lsi2 is electrogenic and no pH measurements have been conducted to show that protons are indeed transported in exchange for Si. The observed effects on Si efflux to changing external pH or the addition of protonophores could have alternative interpretations other than a transport site for protons. For instance, conformational changes to Lsi2 could occur in response to changes in extracellular pH (e.g., via protonation of histidine residues, etc.). Moreover, protonophores can dissipate the ΔpH of a multitude of cellular compartments, including the mitochondrial proton gradient to uncouple respiration. It is also worth noting that, indeed, passive fluxes are also (indirectly) energy-dependent, requiring ATPases to generate the electrochemical gradients that drive these fluxes; thus, pharmacological manipulations of energy gradients do not necessarily and selectively target active-transport mechanisms.
5. Basic kinetic analyses of the Lsi2-mediated Si flux are currently lacking. Although a time course of efflux was performed in OsLsi2-expressing oocytes (see above; Ma et al. 2007), questions pertaining to substrate affinity (K_M_) and velocity (V_max_) of the flux remain unaddressed, as are issues surrounding substrate competition (either direct or indirect; again, see above regarding Ge as a proxy).

Despite these shortcomings, it is nonetheless abundantly clear that Lsi2 is capable of mediating transmembrane Si fluxes. However, improvements are needed to develop a more elaborate and robust model considering some of the inconsistencies listed above. In this context, we discuss and propose approaches aimed at refining and building upon these important findings (see ‘Future directions, hypotheses, and recommendations’, below).

### Protein localization

As Table 1 demonstrates, and unlike Lsi1 that exhibits variable cellular localization patterns across species (spanning epidermis to endodermis), Lsi2 appears confined to the endodermis, at least in the species investigated in the limited number of studies to date. In rice, OsLsi2 is further confined to the proximal (i.e., inward-facing) side of endodermal cells (Ma et al. 2007), whereas in barley, maize, and cucumber, Lsi2 appears to show no polar localization (Mitani et al. 2009b; Sun et al. 2018; Mitani-Ueno and Ma 2020). Unfortunately, our understanding of Lsi2 localization in plants is confined to those species.

These observations have led some to suggest that the ‘coordinated system’ of OsLsi1 and OsLsi2 (i.e., polar localization of the transporters specifically in exo- and endo-dermal cells) may be unique to rice and explain its higher tissue Si content relative to most other species (Ma and Yamaji 2015; Mitani-Ueno and Ma 2020; it is also worth noting that polar localization of transporters also exist for other substrates, such as B, Fe, and auxin; Takano et al. 2010; Barberon et al. 2014; Raggi et al. 2020). It may, however, be premature to draw such conclusions. For one, the typical field conditions for rice (i.e., paddy soils and tropical climate) may be more conducive to soils with higher plant-available Si. Moreover, rice appears to accumulate Si to a very similar extent (when grown under identical conditions) to some species that lack this ‘coordinated system’ or have yet to be characterized as such. For example, Deshmukh et al. (2020) found ‘crookneck pumpkin’ (*Cucurbita moschata*) to accumulate 3.9% leaf Si (and concentrations up to 5% have been reported; Seki and Hotta 1997), contradicting the “passive”/ “intermediate” accumulator designation attributed to this species (Mitani-Ueno and Ma 2020); this despite CmLsi1 being present in all root cell types and without polar localization (Mitani et al. 2011). By contrast, cucumber has been reported to accumulate only 0.15 – 0.20% Si in leaves, despite CsLsi1 being polarly localized at the endodermis (Sun et al. 2017). Lastly, the sunflower species *Helianthus petiolaris* (‘prairie sunflower’; 4.8% leaf Si) and *Helianthus exilis* (‘serpentine sunflower’; 5.2% leaf Si) accumulated more Si than rice (4.7% leaf Si) in controlled experiments (Deshmukh et al. 2020) – the expression/localization pattern of their Si transporters have yet to be determined.

### Physiology

Early physiological studies of Si transport in roots from intact plants have shown that long-term steady-state Si uptake (i.e., net flux) can typically range between 1 – 10 μmol g (root fresh weight)^−1^ h^−1^ (as measured by Si depletion of hydroponic media; Tamai and Ma 2003; Liang et al. 2005; Rains et al. 2006). These rates are indeed consistent with those observed for many mineral nutrients, including calcium (Ca^2+^; Huang et al. 1992), nitrate (NO_3_^−^; Kronzucker et al. 1999), and potassium (K^+^; Coskun et al. 2016).

What remains to be determined, however, are the rates of the unidirectional fluxes of Si (i.e., influx and efflux, separately) and their direction (e.g., towards the xylem apoplast or the rhizosphere), as well as cellular pool sizes (i.e., Si concentration/activity in intracellular compartments, particularly the cytosol and vacuole). The absence of data in these regards may be attributable to methodological constraints, particularly in vivo, such as the unavailability and/or prohibitive cost of Si-selective measurements including long-lived Si radiotracers (e.g., ^31^Si [t_1/2_ = 2.6 h] or ^32^Si [t_1/2_ ≈ 150 y]) and Si-sensitive microelectrodes or fluorescent dyes.

Another question the ‘coordinated’ model raises is how does Si transport proceed from the endodermis to the xylem apoplast: are there unknown Si transporters in pericycle and xylem parenchyma cells, or are we to assume that Si simply travels apoplastically throughout the stele and into the xylem apoplast (see subsection ‘Lsi2 is responsible for xylem loading and tissue Si content’, below)? Within the xylem vessels, NMR analyses have reported Si concentrations up to 8 mM (the vast majority as Si(OH)_4_; Casey et al. 2003; Liang et al. 2005) and as high as 20 mM using calorimetric methods (Mitani and Ma 2005; Nikolic et al. 2007). If such concentrations are indeed representative of xylem Si concentrations in higher plants, they would certainly be consistent with some sort of active transport mechanism to pump Si against what must be a steep uphill concentration gradient.

## Future directions, hypotheses, and recommendations

The previous section summarized the current state of understanding surrounding Lsi2 with respect to its phylogeny, protein structure and function, and physiological link to Si transport *in planta*. We also highlighted where deficiencies may lie in the standing model. Here, we attempt to provide some direction for addressing these gaps in knowledge, which we have organized around three major hypotheses: (1) *Lsi2* expression is regulated by Si, (2) Lsi2 is a secondary active Si(OH)_4_/H^+^ antiporter, and (3) Lsi2 is responsible for xylem Si loading and thus dictates shoot Si content.

### Lsi2 expression is regulated by Si

As Table 1 shows, there is no consensus on how the expression of *Lsi2* (or *Lsi1* for that matter) responds to Si supply. We see contradictory results between studies even within the same species (rice). Indeed, while Ma et al. (2007) showed strong downregulation of *OsLsi2* expression after 3-d Si supply (see also Yamaji and Ma 2011; Mitani-Ueno et al. 2016), Kim et al. (2014) showed a 2- to four-fold *increase* after just 1-d Si supply. In both studies, external Si was set at a concentration of 1 mM, but the japonica cultivars tested were not the same. In maize and barley, *Lsi2* expression was downregulated with Si supply (Mitani et al. 2009b), whereas cucumber *Lsi2* was upregulated (Sun et al. 2018).

Mitani-Ueno et al. (2016) provide an excellent example of how future investigations into *Lsi1* and *Lsi2* gene expression responses ought to be conducted. The authors presented a convincing case that *OsLsi1* and *OsLsi2* expression in rice roots was negatively regulated by shoot Si specifically. Not only did they demonstrate a strong negative correlation between root gene expression and shoot Si content by carefully conducting a dose- and time-dependent growth experiment in WT seedlings, they showed that the loss-of-function *oslsi1* mutant, which accumulated marginal Si in its shoots (0.4% versus 6.1% in WT), did not alter its root expression of *OsLsi1* and *OsLsi2*; interestingly, *OsLsi1* and *OsLsi2* expression was not different between mutant and WT when plants were Si-deplete. A split-root (± Si) experiment revealed that even roots without Si supplementation showed downregulation of *OsLsi1* and *OsLsi2*, further buttressing the hypothesis that a shoot-derived signal was directing gene expression in the roots. Lastly, the authors determined that a specific region of the *OsLsi1* promoter is linked to the gene-expression response; whether this is similarly the case for *OsLsi2* should be investigated.

Whether Lsi2 is regulated at the transcriptional, translational, and/or posttranslational level (e.g., via transcription factors, regulatory proteins, miRNAs, or protein phosphorylation) remains largely unaddressed to date. Recently, a R2R3 MYB transcription factor, OsARM1, was found to negatively regulate genes linked to As(III) transport, including *OsLsi1* and *OsLsi2*, by interacting with the promoter regions and thus regulating the uptake and root-to-shoot translocation of As(III) (Wang et al. 2017). Knocking out *OsARM1* resulted in enhanced As(III) translocation; overexpression resulted in the opposite response. Presumably OsARM1 would similarly regulate Si transport and accumulation; however, this has yet to be demonstrated. Indeed, a vast literature on the regulation of As(III) transport exists, which one could draw upon for research direction (see e.g., Tang and Zhao 2020, and references therein). This includes the involvement of the soluble N-ethylmaleimide-sensitive factor attachment protein receptor (SNARE) protein AtSYP51 in regulating membrane trafficking of AtNIP1;1 (Barozzi et al. 2018), the Ca^2+^-dependent protein kinase AtCPK31 positively regulating As(III) uptake via AtNIP1;1 (Ji et al. 2017), and protein phosphorylation regulating polar localization and endocytosis of AtNIP5;1 (Wang et al. 2017).

It has also been demonstrated that water-deficit stress in general and abscisic acid (ABA) specifically (the phytohormone which accumulates in response to water deficit) can rapidly (within hours) result in downregulation of *OsLsi1* and *OsLsi2* expression and reduce Si uptake (Ma et al. 2006; Yamaji and Ma 2007; 2011). It has been suggested that ABA could directly downregulate *OsLsi1* and *OsLsi2* expression since the promoter regions of both genes display putative ABA-responsive cis-regulatory elements (Yamaji and Ma 2007; 2011); direct evidence of interaction is however forthcoming. Perplexingly, *OsLsi1* expression was shown to be strongly *upregulated* in rice roots in response to salinity stress (50 mM NaCl for 12 d; Senadheera et al. 2009), despite ABA known to accumulate in response to salinity stress, much like with water deficit (Osakabe et al. 2014). Although it remains unclear how to reconcile these reports, the question of *Lsi1* and *Lsi2* gene expression in response to stress is clearly in need of elucidation.

### Lsi2 is a secondary active Si(OH)_4_/ H^+^ antiporter

As outlined in the previous section, much more evidence is required to buttress the widely held model of Si(OH)_4_/H^+^ antiport. Firstly, it is imperative that the concomitant flux be demonstrated, i.e., the H^+^ flux (ΔpH) and its Si-dependence. This can be achieved, for example, by means of pH-sensitive microelectrodes or fluorescent dyes. For instance, pH-sensitive microelectrodes have been routinely used to measure intra- and extra-cellular pH changes in response to fluxes of various substrates, particularly in the *Xenopus* oocyte system (Bröer et al. 1998; Nakhoul et al. 1998; Holm et al. 2005). Likewise, pH-sensitive fluorescent dyes have been traditionally employed to demonstrate H^+^/substrate antiport mechanisms. Using the fluorescence quenching method of pH-sensitive probes such as quinacrine and acridine orange, typically in everted membrane vesicles or reconstituted proteoliposomes, the Na^+^/H^+^ antiport mechanism of transporters such as SOS1 and NHX1 have been routinely characterized and instrumental in understanding plant Na^+^ transport (Apse et al. 1999; Qui et al. 2002), and could help elucidate Lsi2 function. To test the model in vivo, genetically encoded ratiometric pH sensors (pHlourin or pHusion; Gjetting et al. 2012; Martinière et al. 2018) anchored to either side of the plasma membrane can be used to measure changes in apoplastic and cytosolic pH in response to Si.

Other techniques could lend themselves to also further characterizing the Lsi2-mediated Si flux. For example, a novel fluorescent technique utilizing zinc salts to measure Si flux kinetics in SIT-expressing proteoliposomes could be employed to study Lsi2 in vitro (Knight et al. 2016). This technique could also be useful to test alternative hypotheses into whether other co-substrates for Si exist. For example, a suite of cation or anion additions or subtractions from the external media could be performed to test for the existence of other potential co-substrates.

It is also worth considering whether Lsi2 could be an accessory protein to a larger Si-transporter complex. For example, it has been demonstrated that although ArsB can catalyze As(III) efflux coupled to the electrochemical proton gradient, when it binds ArsA, it is converted into a primary ATP-coupled As(III) efflux pump (Garbinski et al. 2019). Alternatively, it could be that Lsi2 function is dependent on unknown cellular constituents which are currently absent in the heterologous expression assay. For example, the plant K^+^ channel AKT1 was only observed to be functional in the *Xenopus* oocyte system once its corresponding protein kinase CIPK23 and Ca^2+^-binding calcineurin B-like proteins CBL1 and CBL9 were co-expressed (Xu et al. 2006). Phosphorylation predictions, such as presented in Fig. 4 (see also Supplementary Fig. S4), could help make inroads towards better understanding this process.

Lastly, we should keep open the possibility that anionic silicate (i.e., some level of deprotonated silicic acid) is the penetrating Si species, at least until such time as this is empirically ruled out. Given the relatively high pKa of Si(OH)_4_ (9.8), one might assume that the penetrating species at physiological pH is of the conjugate acid; however, this may not necessarily be the case. Lsi2 does share some homology with bacterial (poly)anion transporters (see ‘Protein structure’ subsection, above), and thus could potentially provide support for this alternative hypothesis. Moreover, it is worth noting that B efflux is thought to be mediated by BOR transporters in a borate ([B(OH)_4_]^-^)/H^+^ antiport mechanism (pKa of boric acid is 9.24; Miwa and Fujiwara 2010; Onuh and Miwa 2021). Similarly, sizable NH_3 _fluxes were detected in barley roots despite a NH_3_/NH_4_^+^ pKa of 9.25 (Coskun et al. 2013).

### Lsi2 is responsible for xylem loading and tissue Si content

As mentioned above, the question of how Si is transported between the endodermis and xylem apoplast remains elusive but is critical if we are to understand how Si translocation functions and is regulated. Are there Si transporters present in the pericycle and xylem parenchyma that have yet to be discovered, and if so, by what mechanism do they operate? Or are we to presume that Si simply and freely diffuses into the xylem apoplast once released by Lsi2 in the endodermis, as the standing model seems to suggest? One thing is clear, the relative dearth of immunolocalization studies for Lsi2 (see ‘Protein localization’ subsection, above) demonstrates the necessity for many more such analyses. Perhaps then one can get a clearer understanding of the extent of Lsi2 localization.

Once again, we might look to the As(III)-transport literature to shed some light. Indeed, OsLsi2 has been demonstrated to be capable of As(III) transport and a crucial component of the xylem-loading process (Ma et al. 2008), but other transporters have been identified in pericycle and xylem parenchyma cells, including a C-type ATP-binding cassette (ABC) transporter, OsABCC7, which plays a more direct role in xylem loading and root-to-shoot translocation of As(III) (Tang et al. 2019; Tang and Zhao 2020). It is still unknown if such mechanisms exist for Si.

The fundamental role of Lsi2, and whether it evolved to transport Si, or whether it served some other adaptive purpose(s), is also crucial to understand. The fact that *Lsi2* is present in many non-siliceous higher plant species (e.g., throughout the Brassicaceae and Solanaceae; Fig. 2; Coskun et al. 2019a) suggests the latter. However, the question of functionality is crucial: much like with *Lsi1*, until it is verified that the encoded protein is capable of transporting Si, the relevance of the presence/absence of a gene is very limited. Indeed, these genes may have evolved as ancient detoxification mechanisms for substrates such as As(III) or essential micronutrients such as B, and only surreptitiously resulted in Si-derived benefits from also being Si-permeable (in some species).

## Conclusions

A relative lack in the absolute number of studies (including in methodological variety) has left a void where the physiological roles and molecular mechanisms of Lsi2 remain obscure, particularly in comparison to Lsi1. Thus, there is a need for much more supportive evidence to the standing transport model and for additional studies to understand the characteristics of the Si transport cycle. Although it is reasonable to infer an active Si-efflux mechanism, given the passive nature of the influx system and the high concentrations of Si in the xylem and shoot tissues, as it stands, a cautious refrain from perpetuating the claim that Lsi2 is an “active Si transporter” is warranted, until such time as more substantive and direct evidence is presented.

The opportunity to exploit Lsi2 for breeding purposes exists (Bélanger et al. 2016). However, the roles played by this transporter in Si fluxes and the overall physiology of plants need to be elucidated, especially if we are to effectively exploit Si as a prophylactic agent against environmental stress. With increased researcher attention and hypothesis-driven experimentation, Lsi2 need not be a black box in plant Si transport for long.

## Supplementary Information

Below is the link to the electronic supplementary material.

Supplementary file1 (DOCX 1525 kb)

Supplementary file2 (PDF 26 kb)

Supplementary file3 (XLSX 17 kb)

Supplementary file4 (XLSX 36 kb)
