# Supplementary material for: Lsi2: A black box in plant silicon transport
Source: Plant Soil. 2021 Jul 10;466(1-2):1–20. doi: 10.1007/s11104-021-05061-1 (PMC8550040; doi:10.1007/s11104-021-05061-1)
Supplement: Supplementary file 1 — Supplementary file1 (DOCX 1525 kb) [file 11104_2021_5061_MOESM1_ESM.docx]

***
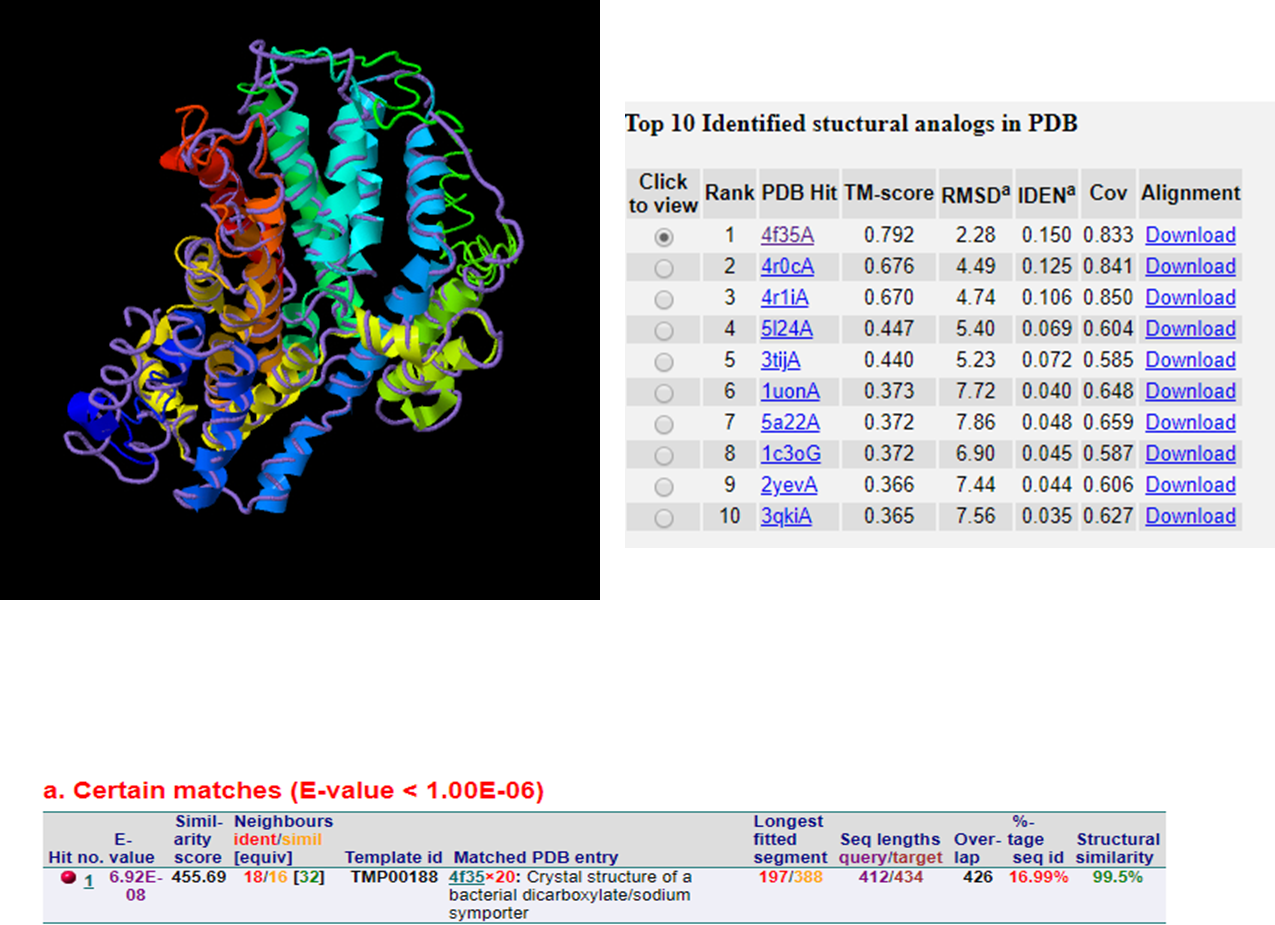
***

**Supplementary Figure S1.** Structural similarity of OsLsi2 with the bacterial dicarboxylate/sodium symporter, NaCT (SLC13A5), according to the I-TASSER algorithm (<https://zhanglab.ccmb.med.umich.edu/I-TASSER/>).

**
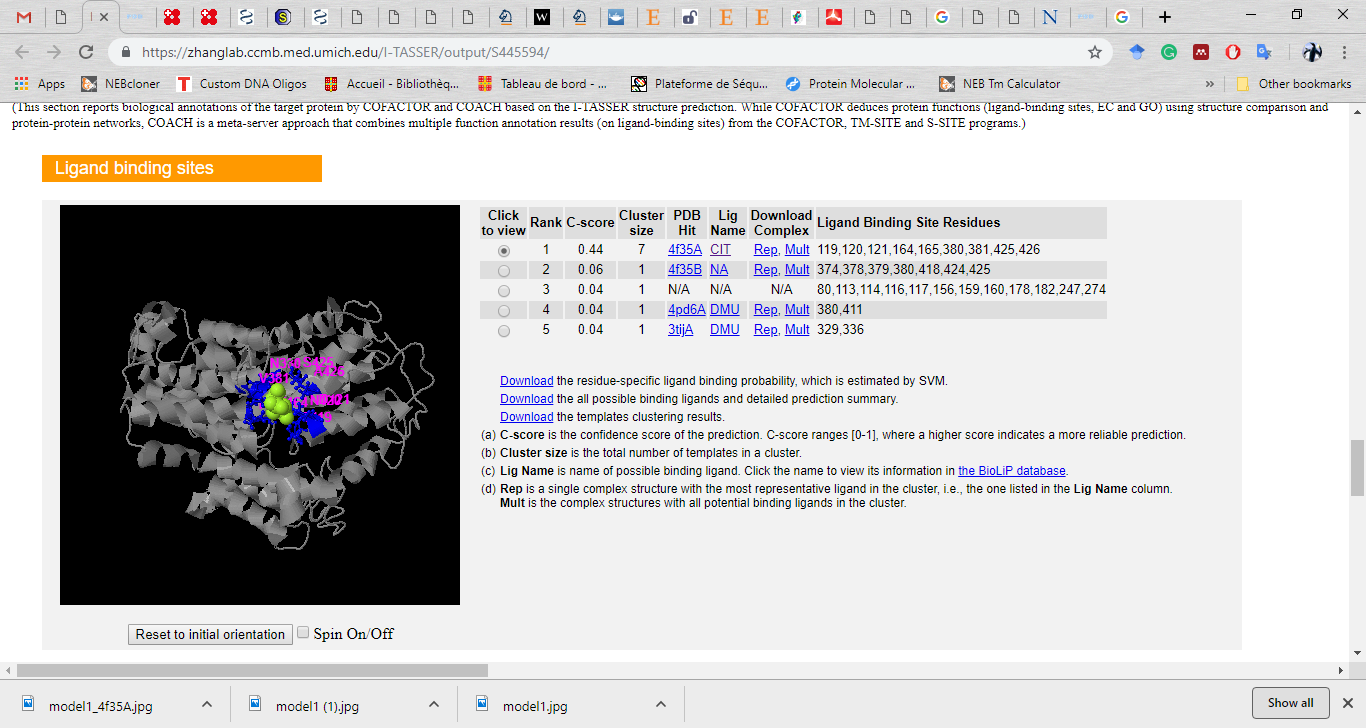
**

**Supplementary Figure S2.** *In-silico* prediction of OsLsi2 ligands and binding sites, according to I-TASSER (<https://zhanglab.ccmb.med.umich.edu/I-TASSER/>). Based on this analysis, citrate is the highest-ranking predicted ligand.


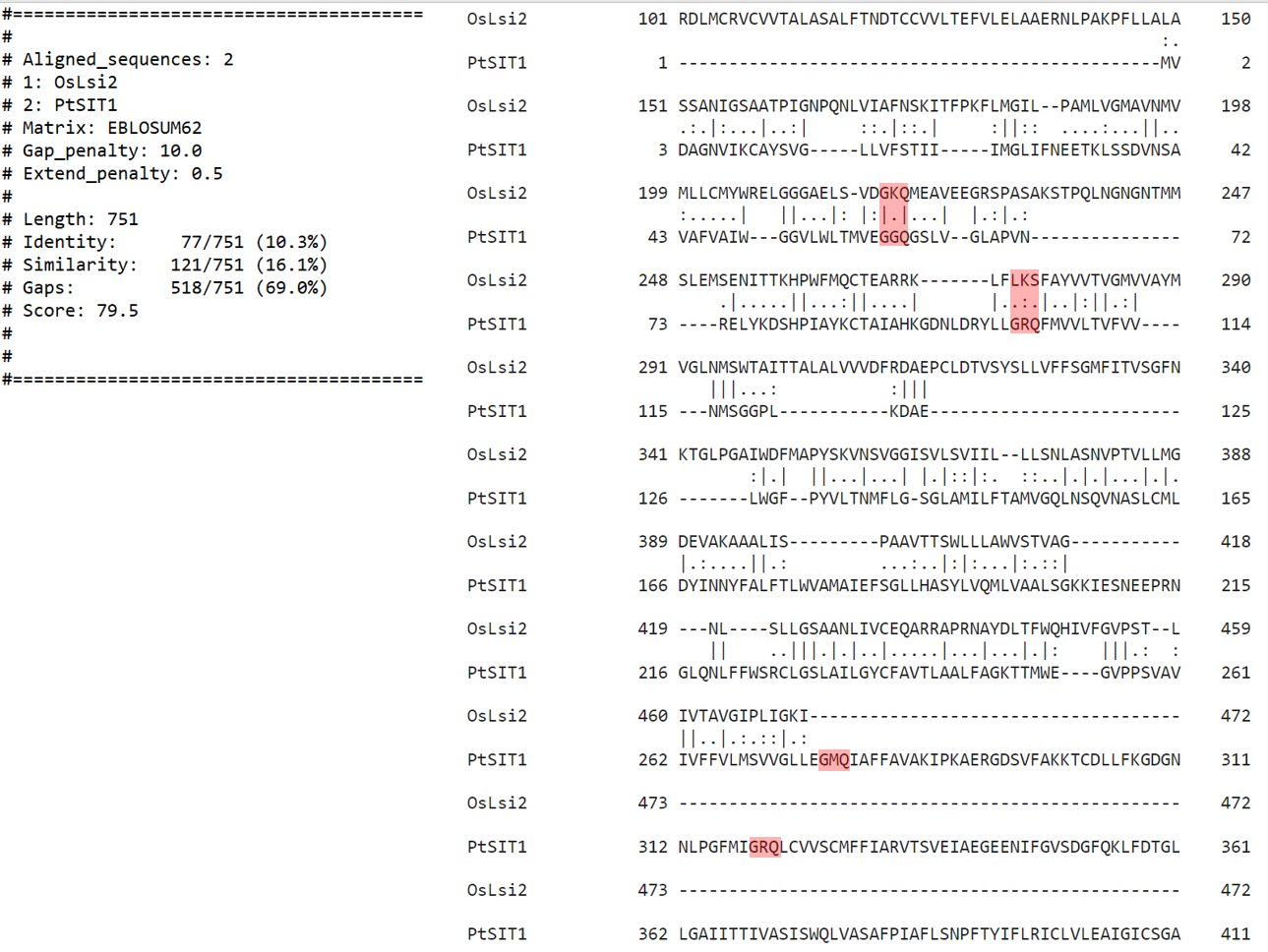


**Supplementary Figure S3**. Pairwise sequence alignment of OsLsi2 (from *Oryza sativa*) and PtSIT1 (from *Phaeodactylum tricornutum*) using EMBOSS Needle (<https://www.ebi.ac.uk/Tools/psa/emboss_needle/>). Highlighted red, the four conserved GXQ motifs thought to dictate Si-selectivity in SIT transporters (Knight et al., 2016). Note, OsLsi2 shares some similarity with the first GXQ pair, but not the second; whether this is related to Si permeability in Lsi2 has yet to be determined.


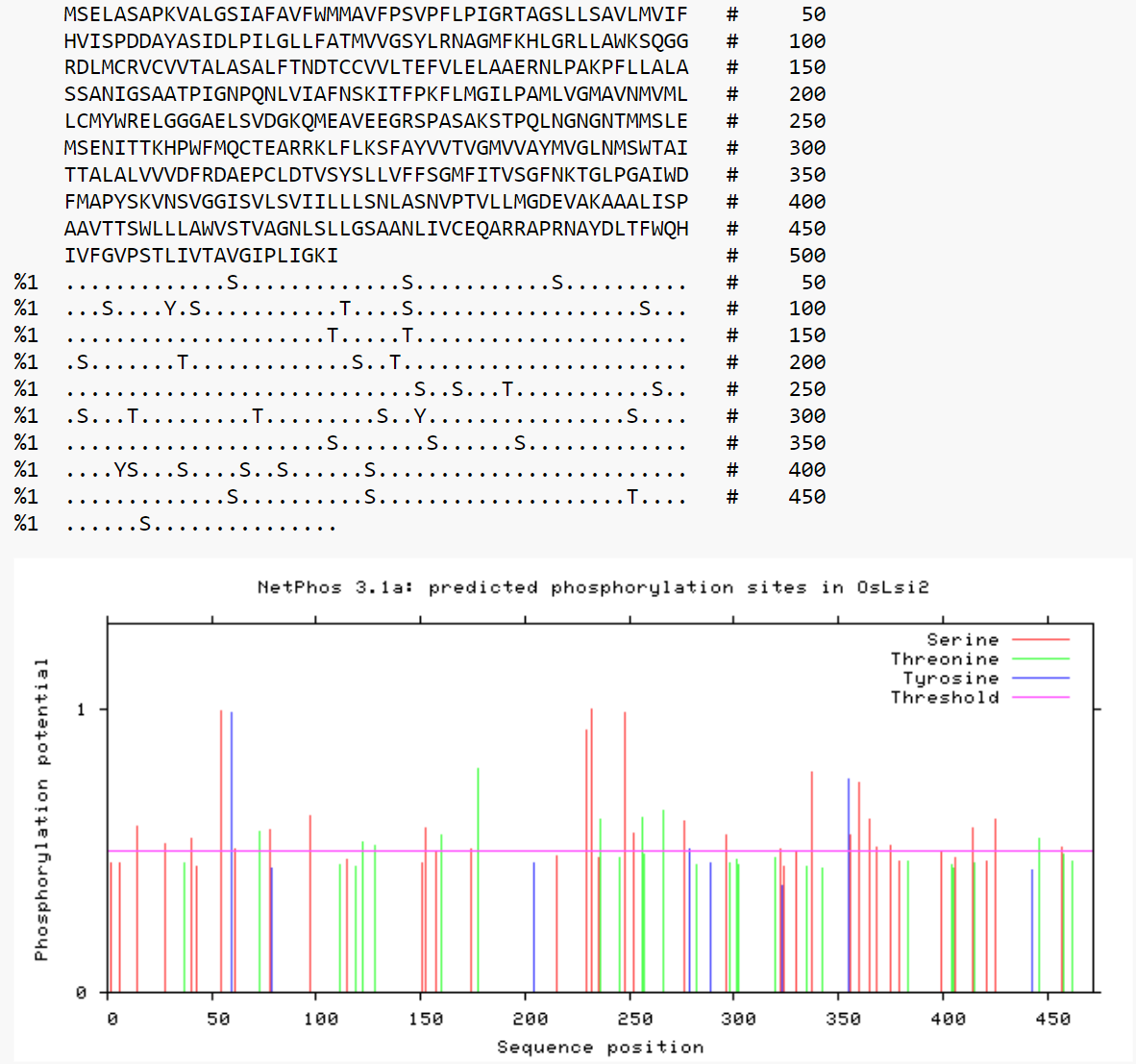


**Supplementary Figure S4.** Predicted phosphorylation sites for OsLsi2. According to the NetPhos 3.1 Server (http://www.cbs.dtu.dk/services/NetPhos/), out of 472 aa, OsLsi2 is predicted to have 38 potential phosphorylation sites.
