# Supplementary material for: Lsi2: A black box in plant silicon transport
Source: Plant Soil. 2021 Jul 10;466(1-2):1–20. doi: 10.1007/s11104-021-05061-1 (PMC8550040; doi:10.1007/s11104-021-05061-1)
Supplement: Supplementary file 2 — Supplementary file2 (PDF 26 kb) [file 11104_2021_5061_MOESM2_ESM.pdf]

Supplementary Table S1. Sequence similarity between OsLsi2 and two other Si transporters from rat (*Rattus norvegicus*) and the diatom *Phaeodactylum tricornutum*. Two other bacterial transporters with predicted structural similarity with OsLsi2 but no reported Si-transport function are also included (SLC13A5 from the bacterium *Vibrio cholera* and arsB from the bacterium *Escherichia coli*; see text for details; see also Supplementary Fig. S1).

**Protein**	**Species**	**# Residues**	**% identity in X residues overlap (relative to OsLsi2)***	**Score***	**Gap frequency***
OsLsi2	Rice (*O. sativa*)	472			
SLC34A2	Rat (*R. norvegicus*)	695	40% in 20	34.0	0%
PtSIT1	Diatom (*P. tricornutum*)	512	23.5% in 34	36.0	0%
NaCT (SLC13A5)	*V. cholera*	449	23.4% in 184	86.0	3.3%
arsB	*E. coli*	429	20.6% in 218	90.0	2.8%

*According to the Expasy SIM Alignment Tool (https://web.expasy.org/sim/).

Supplementary Table S2. Lsi2 secondary structure predictions based on the SOSUI algorithm (https://harrier.nagahama-i-bio.ac.jp/sosui/sosui_submit.html).

**Protein**	**Species**	**Total length (aa)**	**Average hydrophobicity**	**Predicted # TM domains**	**TM6-7 gap (aa)**[**]
OsLsi2	Rice (*Oryza sativa*)	472	0.71	11	71
ZmLsi2	Maize (*Zea mays*)	472	0.79	11	71
HvLsi2	Barley (*Hordeum vulgare*)	474	0.71	11	74
TaLsi2[*]	Wheat (*Triticum aestivum*)	474	0.71	11	74
Monocots (avg ± stdev)		***473 ± 1.16***	***0.73 ± 0.04***		***72.5 ± 1.73***
CmLsi2	Pumpkin (*Curcurbita moschata*)	529	0.46	11	139
CsLsi2	Cucumber (*Cucumis sativus*)	547	0.38	11	157
GmLsi2[*]	Soybean (*Glycine max*)	536	0.56	11	146
AtLsi2[*]	Arabidopsis (*Arabidopsis thaliana*)	502	0.47	11	115
Dicots (avg ± stdev)		***528.5 ± 19.16***	***0.47 ± 0.07***		***139.25 ± 17.78***

* not functionally verified to date; ** number of amino acid residues spanning transmembrane domains 6 and 7; aa, number of amino-acid residues; TM, transmembrane.
